# Glutathione-responsive and -exhausting metal nanomedicines for robust synergistic cancer therapy

**DOI:** 10.3389/fbioe.2023.1161472

**Published:** 2023-03-10

**Authors:** Peng Liu, Lu Hao, Min Liu, Shuo Hu

**Affiliations:** ^1^ Department of Nuclear Medicine, Xiangya Hospital, Central South University, Changsha, Hunan, China; ^2^ Key Laboratory of Biological Nanotechnology, Changsha, China; ^3^ Department of Pharmacy, Xiangya Hospital, Central South University, Changsha, Hunan, China; ^4^ National Clinical Research Center for Geriatric Disorders, Xiangya Hospital, Central South University, Changsha, Hunan, China

**Keywords:** glutathione exhaustion, reduction-response, metal nanomaterials, TME regulation, cancer therapy

## Abstract

Due to their rapid and uncontrolled proliferation, cancer cells are characterized by overexpression of glutathione (GSH), which impairs reactive oxygen species (ROS)-based therapy and weakens the chemotherapeutic agent-induced toxification. Extensive efforts have been made in the past few years to improve therapeutic outcomes by depleting intracellular GSH. Special focus has been given to the anticancer applications of varieties of metal nanomedicines with GSH responsiveness and exhaustion capacity. In this review, we introduce several GSH-responsive and -exhausting metal nanomedicines that can specifically ablate tumors based on the high concentration of intracellular GSH in cancer cells. These include inorganic nanomaterials, metal-organic frameworks (MOFs), and platinum-based nanomaterials. We then discuss in detail the metal nanomedicines that have been extensively applied in synergistic cancer therapy, including chemotherapy, photodynamic therapy (PDT), sonodynamic therapy (SDT), chemodynamic therapy (CDT), ferroptotic therapy, and radiotherapy. Finally, we present the horizons and challenges in the field for future development.

## 1 Introduction

Due to the rapid development of medicine and increasing attention being paid to human health, various therapeutic agents and modalities for combating cancer have begun to be produced. Nanotechnology especially has a prominent position in the area of biomedicine, and nanomedicines are being widely explored for the detection, diagnosis, and treatment of cancer (Yang et al., 2019; Huo et al., 2020). Over the past few decades, metal-based nanomedicines have attracted great scientific interests. Owing to their unique physicochemical and functional properties, metal nanomedicines display enormous potential in a variety of anticancer applications, such as selective drug delivery, tumor microenvironment (TME) regulation, and combination therapy against cancer (Sharma et al., 2015; Hu et al., 2022; Pena et al., 2022).

Tumors have evolved a unique microenvironment, and typically display a more acidic condition, hypoxia, overexpressed reactive oxygen species (ROS), and glutathione (GSH) owing to their rapid proliferation and metabolism (Mura et al., 2013; Li et al., 2019). Metal-based nanomaterials, characterized by degeneration in the TME, can be designed for programmed release of payloads in response to physiological stimulation. This stimuli-responsive behavior can be used for selective drug delivery into tumor tissue or cancer cells, thereby improving therapeutic efficiency and reducing side effects (Zhou et al., 2018; Peng et al., 2022). A typical example is to develop an MnO_2_-based nanocarrier for chemotherapeutics delivery, which could degrade and release drugs in response to overexpressed GSH (Lin et al., 2018).

Interestingly, the TME not only facilitates drug liberation, but could also be manipulated by metal nanomedicines (Cheng et al., 2021). For instance, GSH, composed of cysteine, glycine, and glutamic acid, accounts for the majority of cellular antioxidants to create highly reductive TME and is involved in tumor initiation, progression, and metastasis (Harris et al., 2015; Estrela et al., 2016). The overexpressed GSH endows cancer cells with the capacity to modulate the redox homeostasis and prevents cancer cells from undergoing apoptosis (Franco et al., 2008). Recently, several studies have exhibited that many types of cancer, such as colon, brain, lung, and breast, have significantly increased GSH, and such a change in tumor causes severe resistance and minimized treatment outcomes (Cheng et al., 2021). Therefore, regulation of intracellular GSH level is a promising strategy to boost the efficacy of antitumor treatment, as the exhaustion of antioxidants is highly desirable to sensitize cancer cells to various therapeutic means, such as chemotherapy, photodynamic therapy (PDT), chemodynamic therapy (CDT), and radiotherapy. Meanwhile, several metal nanomedicines are capable of regulating intracellular GSH directly or indirectly, showing their potential in robust antitumor therapy (Zhang et al., 2020a; Liang et al., 2021; Zhao et al., 2022). In the last few years, various GSH modulation-enhanced combinational therapy strategies have been extensively exploited, accompanied by the development of versatile metal-based nanosystems with different structures, functionalities, and treatment performances (Fan et al., 2017).

**SCHEME 1:**
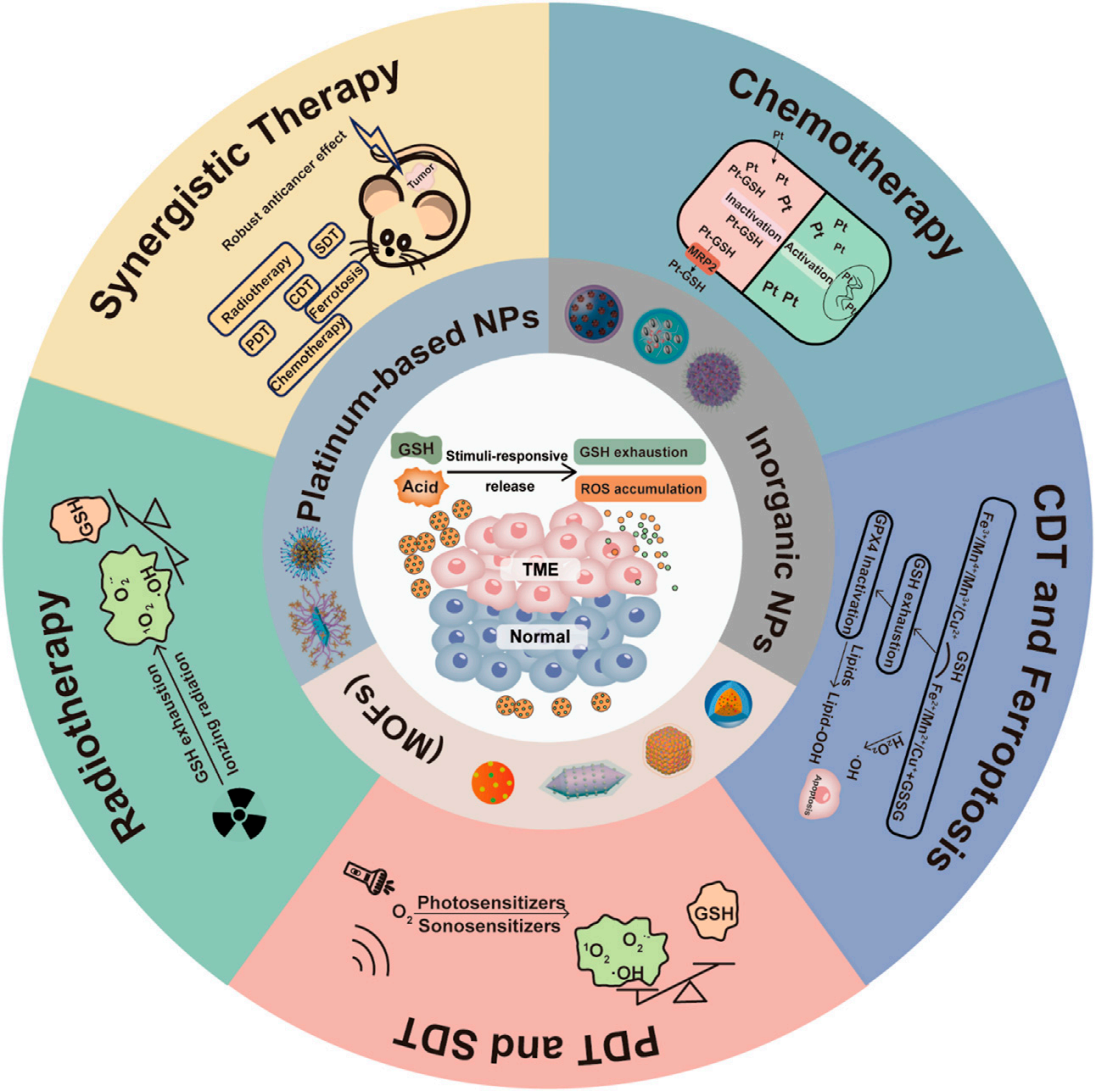
An overview of GSH-responsive and -exhausting metal nanomedicines for synergistic cancer therapy.

In view of the rapid development of GSH-responsive and -exhausting metal nanomedicines in potentiating cancer therapy, we summarize and outline the recent advancements, mechanisms, prospects, and challenges in this field from different strategies (Scheme 1). Firstly, the roles of GSH in cancer cells are introduced to demonstrate the importance of GSH exhaustion in anticancer therapy. Then, we make some classifications of GSH-responsive and -exhausting metal nanomedicines used in cancer therapy, including inorganic nanomaterials, metal-organic frameworks (MOFs), and platinum-based nanomaterials. Subsequently, emphasis will be focused on the GSH-responsive and -exhausting metal nanomedicines for sensitized therapies, such as chemotherapy, PDT, sonodynamic therapy (SDT), CDT, ferroptotic therapy, and radiotherapy. Finally, the horizons and challenges in current and future research areas are also speculated upon.

## 2 The roles of overexpressed GSH in cancer cells

Cancer is characterized by the fast and uncontrolled proliferation of abnormal cells in the body. This unique physiological characteristic of cancer cells needs enough nutrition supply to support their survival and proliferation (Zhang et al., 2020b). For example, in order to maintain intracellular redox homeostasis and build a powerful antioxidant defense, cancer cells are typically characterized by high level of antioxidants, especially GSH with a high concentration of ∼10 mM, which is approximately ten times higher than that in normal cells (Forman et al., 2009). The overexpressed GSH, in the form of cellular free thiols, can generate highly reductive TME to resist DNA damage and protein homeostasis disorder, and protect tumor cells from programmed cell death (Labarrere and Kassab, 2022).

In general, elevated GSH can be observed in many cancers, including lung cancer, ovarian cancer, breast cancer, and head-neck cancer, and this adaptive upregulation of GSH has various effects on tumor advancement, metastasis, and drug resistance (Hu and Liu, 2020). In terms of promoting tumor progression, studies showed that GSH can affect cell apoptosis by regulating the antiapoptotic protein of Bcl-2 family and Caspase activity (Luis Garcia-Gimenez et al., 2013). Metastasis is also associated with GSH in malignant tumors. For example, as a member of the mitochondrial transporter family (SLC25), SLC25A22 can promote glutamate to enter the mitochondrial matrix, thus accelerating the GSH synthesis process. Meanwhile, the gene level of matrix metallopeptidase nine and tumor necrosis factor α related to tumor metastasis exhibits a downward trend in SLC25A22-silenced cancer cells, indicating inhibition of GSH synthesis may play a critical role in the treatment of cancer metastasis (Wong et al., 2016). Finally, the increase of GSH is related to the drug resistance of tumor cells. GSH could detoxify heterogeneous substances under the catalysis of Glutathione S-transferases (GST), then secrete them *via* the multidrug resistance associated proteins (MRPs) efflux pump (Kuermanbayi et al., 2022). Therefore, the overexpression of GSH is considered to be one of the potential causes of tumor’s resistance to various therapeutic approaches.

## 3 GSH-responsive and -exhausting metal nanomaterials

### 3.1 Inorganic nanomaterials

**FIGURE 1:**
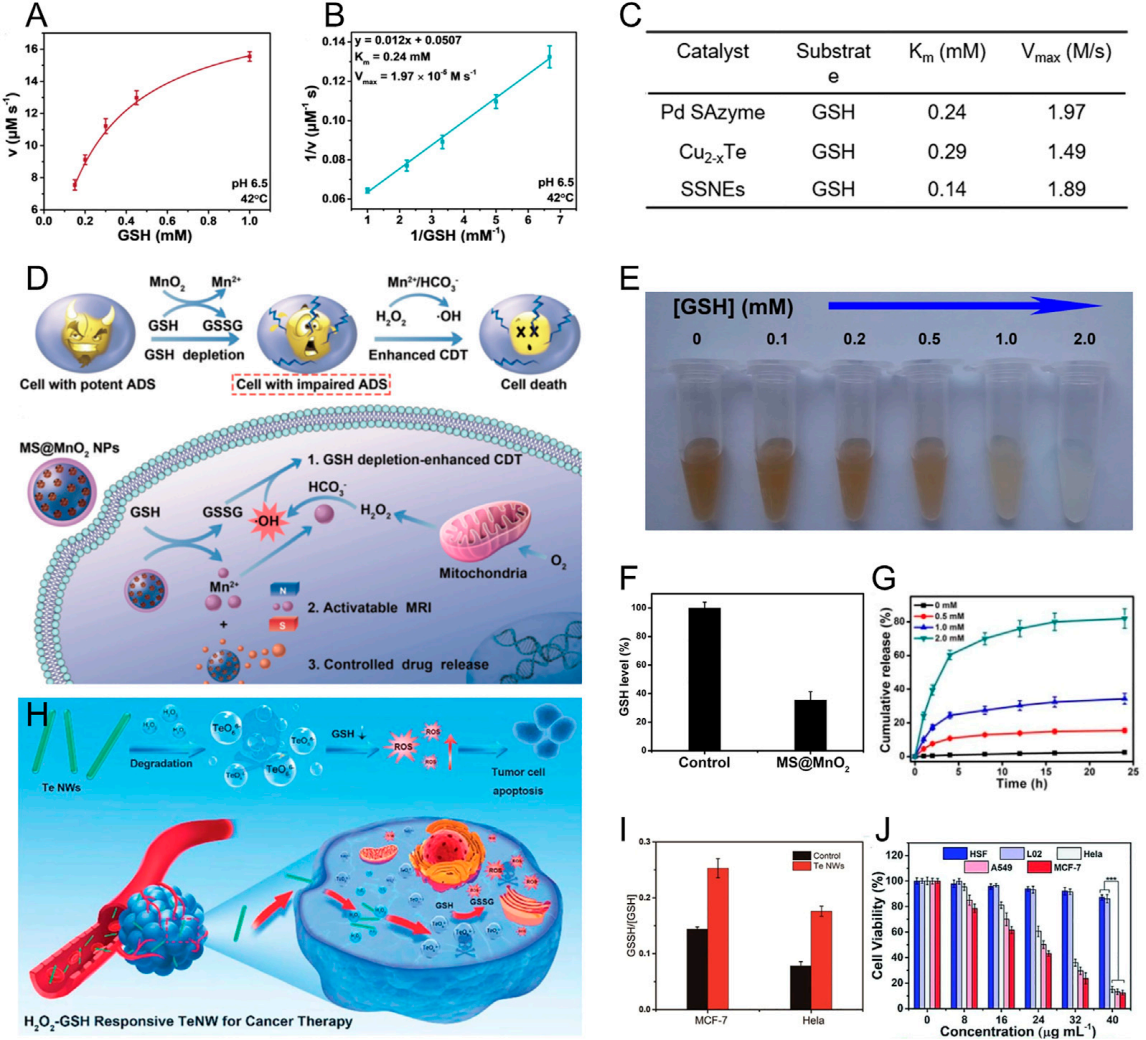
**(A)** Michaelis-Menten kinetics and **(B)** Lineweaver-Burk curves for Pd SAzyme with GSH as a substrate. **(C)** The K_m_ and V_max_ of as prepared Pd SAzyme, Cu_2-x_Te, and SSNEs with GSH as the substrate for GSH peroxidase catalysis. Reproduced with permission. Copyright 2021 John Wiley & Sons. **(D)** The mechanism of MnO_2_ for Fenton metal ion delivery and GSH exhaustion. **(E)** Photo of MnO_2_-MSNs after incubating with different concentrations of GSH. **(F)** The GSH level of U87MG cells after the incubation with MnO_2_-MSNs. **(G)** Release behavior of CPT from GSH-treated MnO_2_-MSNs. Reproduced with permission. Copyright 2018 John Wiley & Sons. **(H)** Schematic illustration of the H_2_O_2_/GSH-responsive TeNWs for selective cancer therapy. **(I)** Histogram of the ratio of GSSH/GSH in MCF-7 and Hela cells after being treated with TeNWs. **(J)** Anticancer activity of TeNWs against cancer cells (Hela, A549, MCF-7) and normal cells (HSF, L02). Reproduced with permission. Copyright 2019 Royal Society of Chemistry.

Inorganic nanomaterials that have intrinsically unique physicochemical properties and can provide satisfactory functionality show enormous potential in biomedical applications, especially in cancer therapy (Wang et al., 2021a). For example, several inorganic nanomaterials, including metal chalcogenide nanoparticles, hybrid metal nanoparticles, and single-atom metal nanoparticles, can act as GSH peroxidase-mimicking nanozymes to exhaust intracellular GSH in cancer cells (Ferguson and Bridge, 2019; Chang et al., 2022). In this process, the nanozyme can catalyze GSH into glutathione disulfide (GSSG) by oxidation of H_2_O_2_, realizing tumor-specific catalytic therapy with response to the TME featuring overexpressed GSH and H_2_O_2_ (Yin et al., 2019). As a typical example, Yang et al. designed PtCu_3_-PEG nanocages, which can serve as horseradish peroxidase-like and GSH peroxidase-like nanozymes for enhanced ROS-based therapy by exhaustion of GSH (Zhong et al., 2020). The rapid and continuous depletion of GSH was observed upon adding H_2_O_2_ into the PtCu_3_-PEG nanocages solution. Afterwards, significant decrease of GSH and elevation of ROS in 4T1 cells were monitored after PtCu_3_-PEG and ultrasound irradiation treatment. It is interesting that some single-atom metal nanozymes showed stronger GSH peroxidase catalytic activity owing to their large portion of superficial active-site atoms and high atomic utilization efficiency (Kaiser et al., 2020). Lin and co-workers rationally designed a single-atom Pd nanozyme (Pd SAzyme) with atom-economical utilization of catalytic centers (Figure 1A); (Chang et al., 2021) the Pd SAzyme displayed Michaelis-Menton kinetics during the catalytic reaction of GSH (Figures 1B, C). They also compared the catalytic rate of GSH reduction induced by single-atom Pd nanozyme, Cu_2-x_Te nanozyme, and copper hexacyanoferrate nanozyme (SSNEs) (Figure 1D), (Wen et al., 2019; Wang et al., 2021b) and first reported that Pd-based nanozyme can imitate GSH peroxidase with robust catalytic activity.

The transition of metal ions valence would also be a powerful strategy for GSH exhaustion. Several metal-based nanoparticles harboring multivalent elements, such as Cu^2+^/Cu^+^, Mn^4+/3+^/Mn^2+^, Mo^6+^/Mo^4+^, and Ir^4+^/Ir^3+^, possess strong oxidization, and can directly react with GSH (Cheng et al., 2021; Dong et al., 2021; Nie et al., 2022). For example, copper ions can lead to GSH depletion through two mechanisms: the direct downregulation *via* the redox reaction between Cu^2+^ and intracellular GSH, and the activation of a Fenton-like reaction by generating Cu^+^ to amplify oxidative stress (Dong et al., 2020). Therefore, Cu-based nanomaterials have been extensively utilized as essential nanomedicines against cancer. Zhang et al. designed biomimetic copper/manganese silicate nanospheres (mCMSNs) to achieve GSH-eliminated synergistic therapy, where *in situ* GSH in tumor triggered mCMSNs biodegradation and reduced Cu^2+^ into Cu^+^ for enhanced hydroxyl radical production, leading to a powerful inhibition effect on tumors (Liu et al., 2019). In addition, manganese oxide nanomaterials (MONs), including MnO_2_, Mn_2_O_3_, and Mn_3_O_4_, possess a similar GSH elimination property to Cu-based nanomaterials, and react with GSH along with the decomposition of MnO_x_ into Mn^2+^ for initiation of the Fenton-like reaction (Ding et al., 2020; Qian et al., 2020). Chen and co-workers first presented the MnO_2_-based nanoagent with both Fenton metal ion delivery and GSH exhaustion capability (Figure 1E) (Lin et al., 2018). This nanoagent could be decomposed by GSH to create Mn^2+^ and GSSG, accompanied by the elimination of intracellular GSH (Figure 1F). The generated Mn^2+^ displayed Fenton-like activity for enhanced CDT. Furthermore, the authors validated the capacity of MnO_2_-coated mesoporous silica nanoparticles (MSNs) to control drug release, and the camptothecin (CPT)-loaded MnO_2_-MSNs showed sustained CPT release after the addition of GSH (Figure 1G). This GSH-responsive behavior not only caused contrast-enhanced magnetic resonance imaging, but also contributed to the tumor site-specific release of loaded therapeutic agents for realizing selective cancer therapy (Klochkov et al., 2021; Wu et al., 2022). Therefore, in view of the potential application in drug delivery and GSH depletion, MONs are an important choice for oxidative stress-amplified synergistic therapy.

More interestingly, several semimetallic nanomaterials, such as selenium (Se)- and tellurium (Te)-based nanoparticles, can also decrease intracellular GSH levels. Yang et al. designed manganese-doped VSe_2_ nanosheets for hyperthermia-assisted tumor therapy by dual depleting the GSH (Zhao et al., 2022). The Se-Se bonds in the nanosheets can interact with GSH, which synergizes with the valence states transition of manganese ions, resulting in enhanced reduction of GSH levels. Collectively, this dual exhaustion effect significantly boosts •OH production and improves therapeutic effect. Inspired by the unique characteristic of Se-based nanomaterials, the Te with the similar chemical properties to Se was developed for selective cancer therapy by Yang and co-workers (Wu et al., 2019). They synthesized inorganic tellurium nanowires (TeNWs), which produce toxic TeO_6_^6-^ under intracellular overexpressed H_2_O_2_ condition and boost enhanced ROS generation in the tumor site. Meanwhile, the formed TeO_6_^6-^ depletes the intracellular GSH and forms GSSG (Figure 1H). The ratio of intracellular GSSG/GSH was obviously elevated after MCF-7 and HeLa cells were treated with TeNWs (Figure 1I). Owing to the overproduced H_2_O_2_ and GSH in tumor cells, TeNW can selectively eliminate cancer cells while normal cells survive (Figure 1J).

### 3.2 Metal-organic frameworks

**FIGURE 2:**
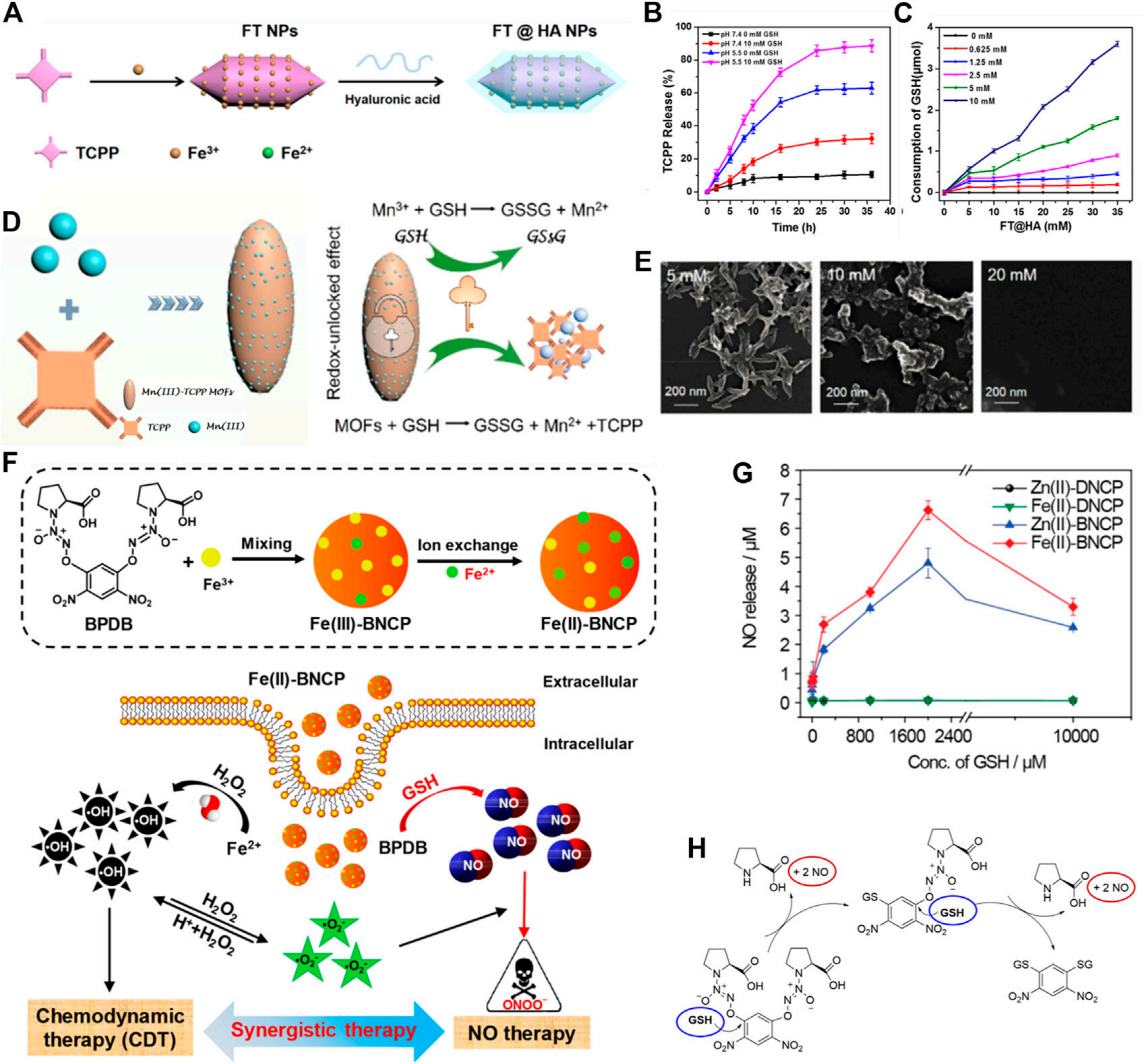
**(A)** Schematic illustration of FT@HA synthesis. **(B)** The pH and GSH-responsive TCPP release. **(C)** Detection of GSH consumption. Reproduced with permission. Copyright 2020 American Chemical Society. **(D)** The scheme of Mn^3+^-TCPP MOFs preparation and mechanism for redox-unlocked therapy. **(E)** Scanning electron microscope images of Mn^3+^-TCPP MOFs with various concentrations of GSH. Reproduced with permission. Copyright 2019 American Chemical Society. **(F)** Schematic illustration of Fe(II)-BNCP preparation and NO-CDT synergistic therapy. **(G)** NO release from Fe(II)-BNCP at various levels of GSH. **(H)** The mechanism of NO release from Fe(II)-BNCP in high levels of GSH. Reproduced with permission. Copyright 2019 American Chemical Society.

The MOFs, assembled from metal ions and organic ligands *via* coordination bonds, have attracted growing interest in biomedical applications owing to their biocompatibility, biodegradability, adjustable structures, and diverse functions (Liang and Liang, 2020; Ni et al., 2020). After rational design, MOFs can be endowed with intrinsic GSH-responsiveness and depletion capability. As a typical example, TCPP-based MOFs (TCPP: tetrakis (4-carboxyphenyl) porphyrin) are recognized as prospective nanophotosensitizers, such as Fe-TCPP, Mn-TCPP, and Cu-TCPP, which could be decomposed in overproduced GSH conditions, leading to intracellular GSH elimination (Wan et al., 2019; Wang et al., 2019; Wang et al., 2020a). Such MOFs have been exploited as GSH consuming-enhanced PDT agents for enhanced cancer therapy. For instance, Fan et al. prepared TME-responsive Fe-TCPP MOFs with hyaluronic acid (HA) encapsulation, named FT@HA (Figure 2A) (Wang et al., 2020a). The FT@HA nanoparticles were rapidly disassembled under acidic and GSH-overexpressed microenvironments, and then released Fe^3+^ and TCPP. The released Fe^3+^ exhausted intracellular GSH *via* Fenton reaction, boosting ROS production by TCPP-mediated PDT (Figures 2B, C). Similarly, Mn has also been utilized in the fabrication of TCPP-based MOFs to consume the intracellular GSH. Zhang et al. reported a MOF nanosystem based on coordination between TCPP and Mn^3+^ for selective tumor damage by redox-unlocked effect (Figure 2D) (Wan et al., 2019). This MOF nanosystem could react with GSH to generate Mn^2+^, TCPP, and GSSG, leading to decomposition of MOF. The morphology of the MOF nanosystem was seriously disintegrated with a rise in GSH concentration (Figure 2E).

In addition to using a photosensitizer as a ligand to construct the MOFs-based GSH-responsive and -exhausting nanomedicines, a modified ligand by a GSH-sensitive linker has also been proposed. Ding et al. designed nanoscale MOFs coordinated by GSH-sensitive NO donor (termed BPDB) and Fe^3+^, named Fe(II)-BNCP (Figure 2F) (Hu et al., 2019). The overexpressed GSH in cancer cells would degrade the framework of Fe(II)-BNCP, and trigger rapid NO liberation *in situ*, thereby leading to synergistic NO-CDT effect for the retardation of tumor growth. The authors found that BPDB-based MOFs exhibited GSH concentration-dependent NO release (Figure 2G), and four molecules of NO can be released from each BPDB molecule provoked by two molecules of GSH (Figure 2H). In another example, Zhao et al. synthesized a kind of MOFs using Zn^2+^ and disulfide-containing imidazole for Ce6 delivery (Meng et al., 2019). Such MOFs can be disassembled under high levels of GSH condition and released Ce6, meanwhile controlling the extent of GSH exhaustion through the disulfide-thiol exchange reaction. Compared with the disulfide-free MOFs, the prepared all-active MOFs showed remarkable GSH-exhaustion capability, contributing to the cancer elimination in PDT.

### 3.3 Platinum-based nanomaterials

Platinum drugs, including cisplatin, oxaliplatin, and carboplatin, play an indispensable role in current cancer treatments. However, their therapeutic outcomes are limited because of drug resistance due to the intracellular inactivation effect (Johnstone et al., 2016). GSH was recognized as a major cellular detoxification agent by forming a Pt (GS)_2_ complex with platinum drugs. This complex can then be cleared by MRPs (Kilari et al., 2016; Cheng and Liu, 2017). To overcome these problems, the multifunctional Pt (IV) prodrugs with excellent stability and superior therapeutic efficacy were developed. After uptake by cancer cells, Pt (IV) prodrugs are reduced by intracellular GSH to produce active Pt (II) complexes, leading to the exhaustion of GSH. As a result, the inactivation of platinum drugs was obviously relieved, which has significant therapeutic effects towards platinum drug-resistant cancer (Ling et al., 2019). For example, Zhang et al. synthesized GSH-consuming nanoparticles using a reduction-sensitive amphiphilic polymer (P1), and encapsulated Pt (IV) prodrugs within the nanoparticles (Wang et al., 2021c). Such a nanosystem could activate cisplatin by exhausting the intracellular GSH, which elevated the Pt-induced DNA intrastrand or interstrand crosslinks, thereby contributing to the anticancer efficiency of platinum drugs.

**FIGURE 3:**
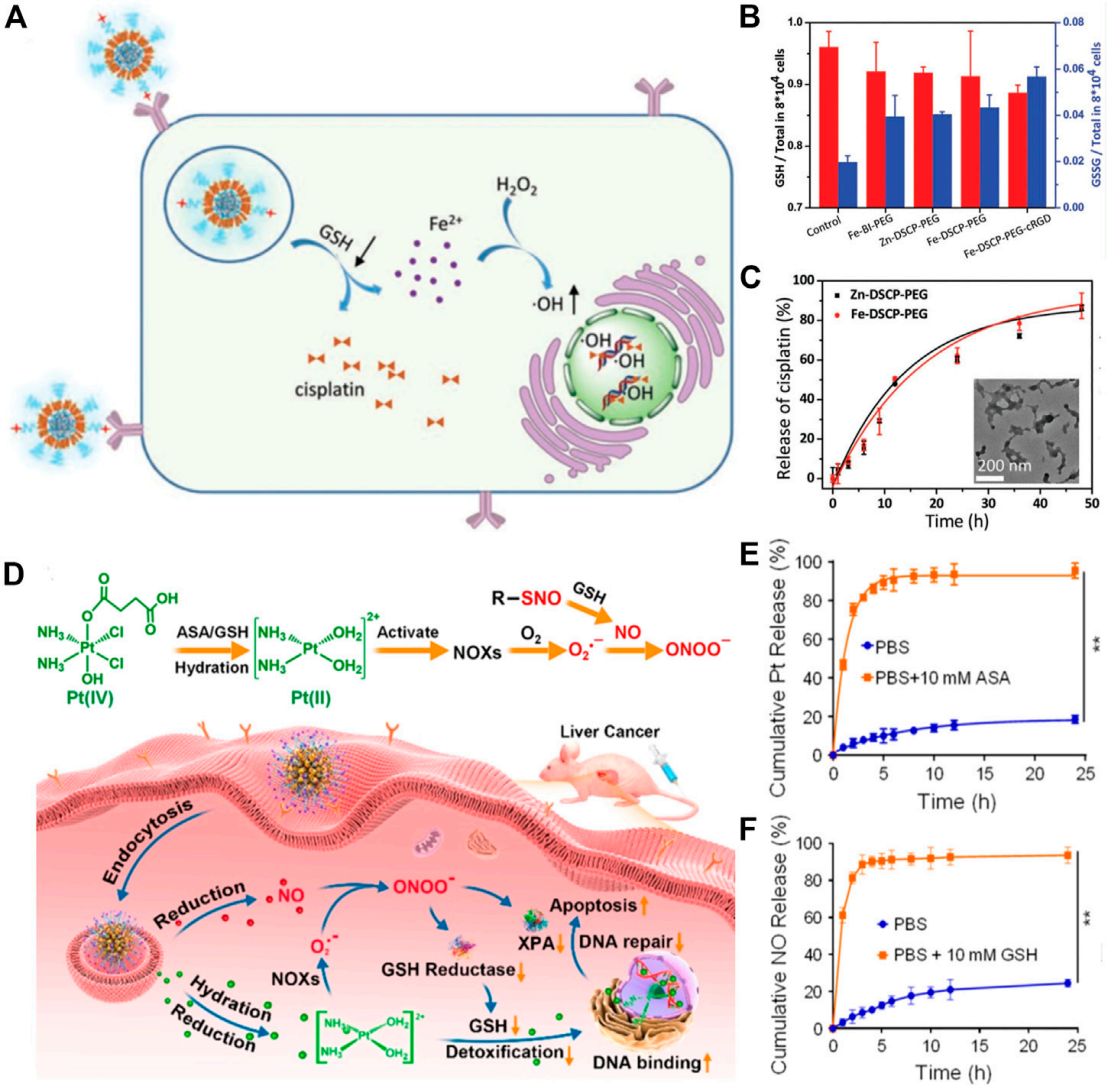
**(A)** Illustration showing GSH consumption induced Fe^3+^ and active cisplatin release, and ROS-mediated DNA damage. **(B)** The GSH and GSSH contents in C6 cells after performing various treatments. **(C)** The cisplatin release behavior and transmission electron microscope image of various nanoscale coordination polymers after treatment with GSH. Reproduced with permission. Copyright 2020 American Chemical Society. **(D)** Schematic illustration of ONOO^−^ generation by SPNA for enhanced Pt-based chemotherapy. **(E)** Pt and **(F)** NO release behaviors of SPNA in PBS with or without ASA/GSH (ASA is a reductive substance similar to GSH). Reproduced with permission. Copyright 2021 American Chemical Society.

Moreover, Pt (IV) complexes have been intensively exploited in the development of GSH-responsive platinum drugs for controlled intracellular drug release. Liu and co-workers developed a biodegradable nanoscale coordination polymer (termed Fe-DSCP-PEG-cRGD), which was self-assembled from Fe^3+^ and cisplatin prodrug, and modified with PEG-cRGD (Liu et al., 2020). The Fe-DSCP-PEG-cRGD could react with overproduced GSH in cancer cells to release Fe^3+^ and active cisplatin; meanwhile, the produced Fe^3+^ was reduced by GSH into Fe^2+^, accompanying the generation of GSSG (Figures 3A, B). The authors validated that cisplatin could be gradually liberated from Fe-DSCP-PEG-cRGD with time and the structure of nanoparticles was largely collapsed (Figure 3C). Similarly, Jin et al. developed supramolecular prodrug nanoassemblies (SPNA) for reactive nitrogen species (RNS)-enhanced chemotherapy (Figure 3D) (Deng et al., 2021). The endogenous GSH could trigger the release of NO and active Pt (II) from SPNA simultaneously (Figures 3E, F), thereby generating highly toxic peroxynitrite anion (ONOO^−^). The continuous production of ONOO^−^ could suppress the activity of glutathione reductase by nitration modification, thus collectively reducing detoxification and blocking DNA damage repair.

## 4 Robust synergistic therapy against cancer

### 4.1 Sensitized chemotherapy

As a chemotherapeutic agent, platinum is one of the most widely used metal-based anticancer components (Ghosh, 2019). The main mechanism of platinum drugs is to hamper the replication and transcription of DNA by forming covalent adducts between platinum complexes and subcellular compounds like DNA, RNA, and other proteins (Amable, 2016). Unfortunately, tumor cells are only susceptible to chemotherapy initially. With the development and recurrence of disease, tumor cells gradually emerged platinum-based resistance. The mechanisms are multifactorial such as the function of nucleotide excision repair pathway, decreased intake as well as increased efflux of platinum drugs, and, medicine inactivation associated with GSH or metallothioneins, which is the most crucial reason for high-level drug resistance (Makovec, 2019). More interestingly, it was discovered that cisplatin-resistant cells manifested high levels of GSH, accompanied by accelerated accumulation of MRPs (Pearson and Cowan, 2021). Meanwhile, GST catalyzes the formation of platinum-GSH compound and accelerates drug inactivation *via* increasing drug solubility (Rabik and Dolan, 2007). Recently, exhaustion of GSH in cisplatin-resistant cells has been extensively utilized to strengthen the cytotoxicity of cisplatin and sensitize chemotherapy (Byun et al., 2005).

**FIGURE 4:**
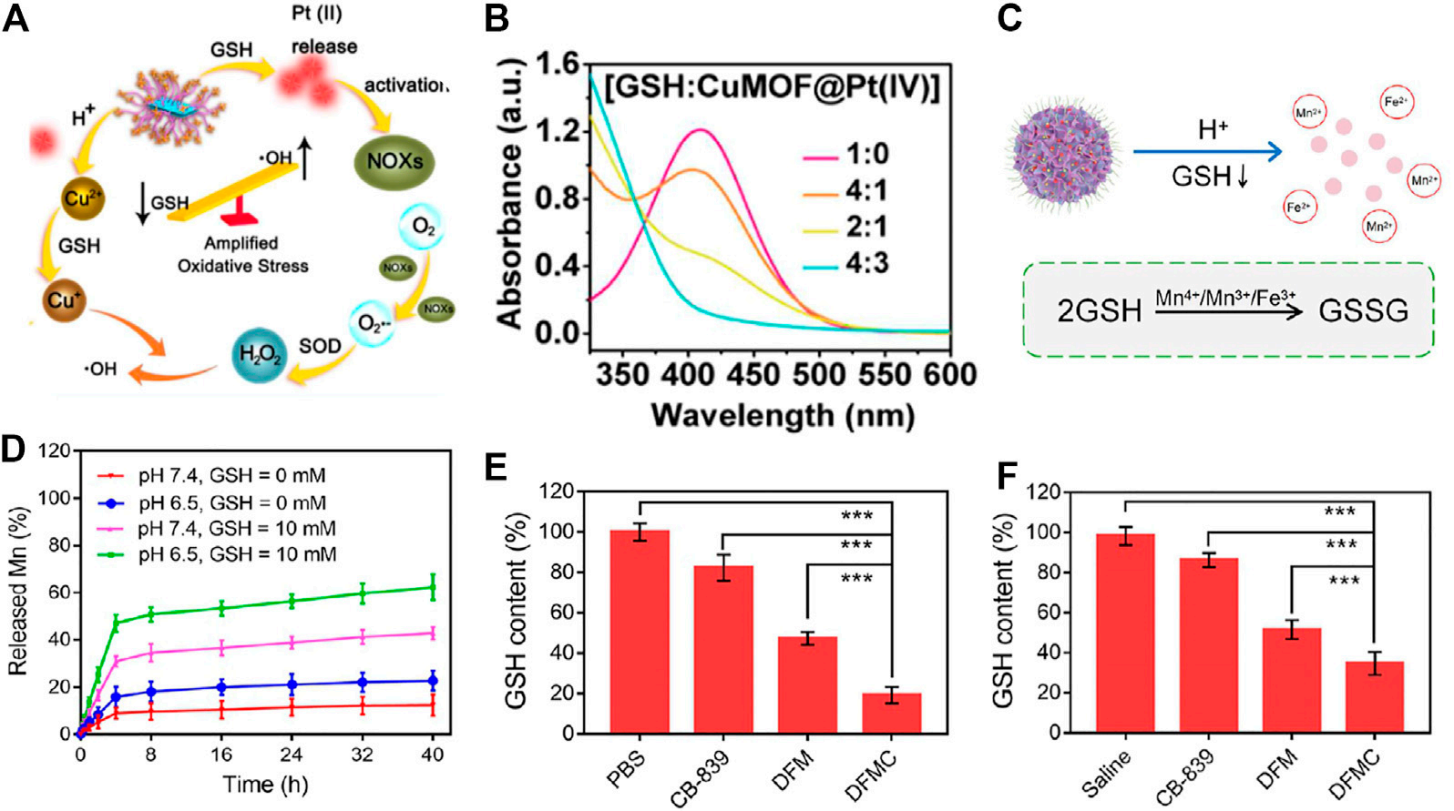
**(A)** Schematic illustration of GSH consuming-sensitized chemotherapy. **(B)** The capability of depleting GSH with CuMOF@Pt (IV) using 5, 5′-dithio-bis-2-(nitrobenzoic acid) (DTNB) as an indicator. Reproduced with permission. Copyright 2021 Elsevier. **(C)** DFMC was decomposed in an acidic environment and GSH was oxidized to GSSG *via* reacting with Mn^4+^/Mn^3+^/Fe^3+^. **(D)** Release state of Mn ions in the presence or absence of GSH with different pHs. **(E)** The level of GSH in CT26 cancer cells and **(F)** tumor tissue in the treatment of PBS, CB-839, DFM (DFMC without CB-839) and DFMC groups. Reproduced with permission. Copyright 2022 American Chemical Society.

Platinum precursors such as Pt (Ⅳ) are applied to produce Pt (Ⅱ) as a GSH-scavenging agent. Chen et al. designed a Pt (Ⅳ)-based responsive nanoreactor modified by Cu^2+^-based MOF (termed CuMOF@Pt (Ⅳ)) for enhanced chemotherapy *via* depleting GSH (Xiang et al., 2021). After being internalized by tumor cells, CuMOF@Pt (Ⅳ) disintegrated in a moderately acidic environment and released Cu^2+^ and Pt (Ⅳ). Under the stimulation of overexpressed GSH, Cu^2+^ and Pt (Ⅳ) were reduced to Cu^+^ and Pt (Ⅱ), respectively (Figure 4A). This redox reaction triggered by GSH conspicuously reduced the inactivation and efflux of cisplatin and enhanced its chemotherapy toxicity, resulting in considerable inhibition on tumor cells. The authors also confirmed that, after mixing different concentrations of CuMOF@Pt (Ⅳ) with GSH, the GSH was depleted significantly with the accumulation of CuMOF@Pt (Ⅳ), indicating its potential consuming-enhanced anticancer ability (Figure 4B). In addition, GSH-responsive platinum agents have also been exploited to endow Pt-based drug sensitization capability. Yang and co-workers reported a nanomedicine composed of dendritic mesoporous silica nanoparticles (DMSNs), Fe_3_O_4_, Mn, and glutaminase inhibitor Telaglenastat (CB-839) (termed DFMC) for restored oxaliplatin chemosensitivity on the basis of the GSH-responsive and -exhausting effect (Figure 4C) (Wu et al., 2022. Interestingly, DFMC collapsed rapidly after being endocytosed in tumor cells, displaying a noticeably responsive-released behavior in the presence of GSH and an acidic microenvironment (GSH = 10 mM, pH = 6.5) (Figure 4D). Simultaneously, released high-valent ions like Mn^4+^/Mn^3+^/Fe^3+^ underwent a redox reaction with intracellular overproduced GSH. Depleted GSH worked together with the glutaminase inhibitor to relieve oxaliplatin efflux, resulting in remarkable anticancer efficiency in tumor cells and tissue (Figures 4E, F).

### 4.2 Enhanced CDT and ferroptotic therapy

**FIGURE 5:**
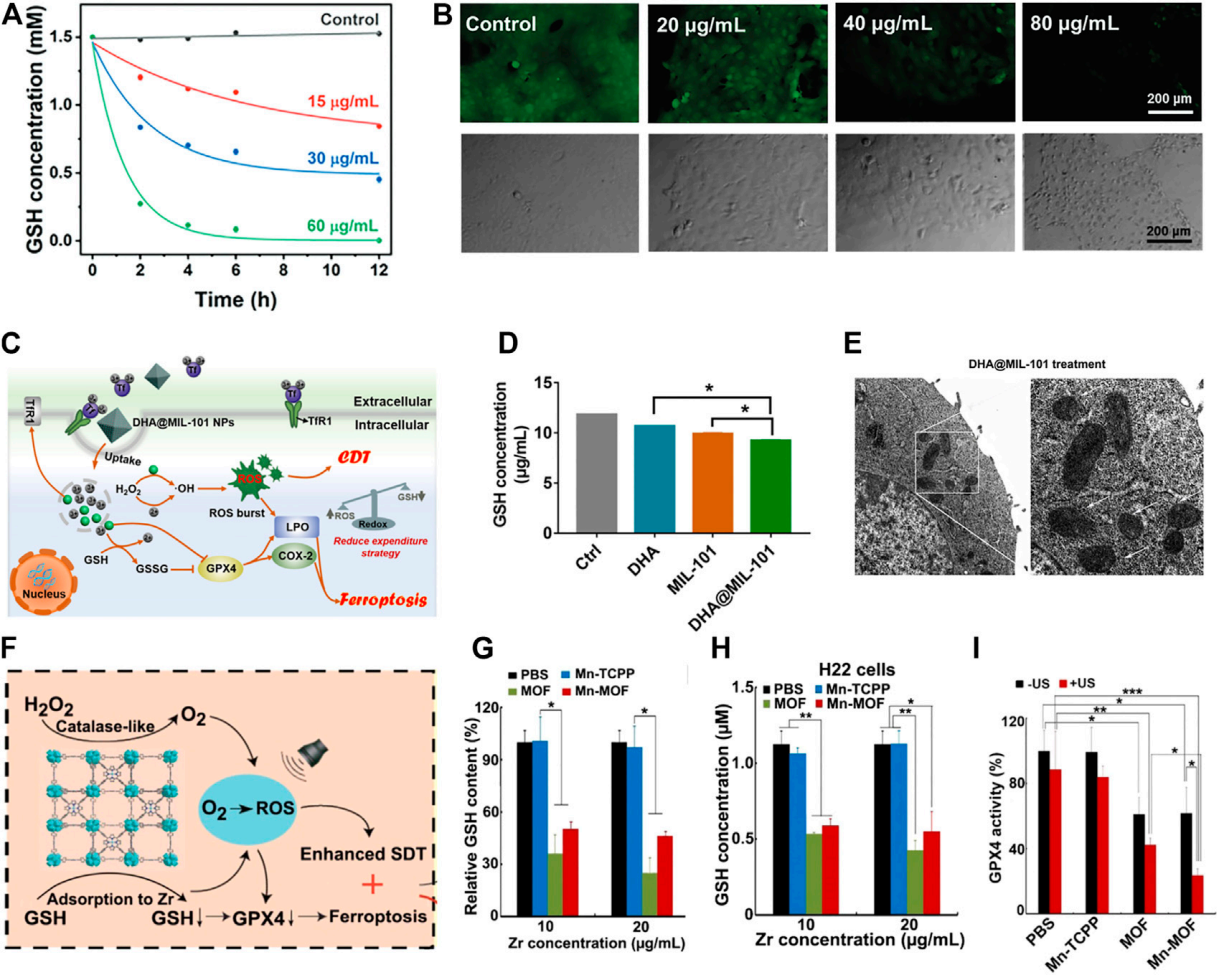
**(A)** Investigation of the capacity of CCF with different concentrations to exhaust GSH at different time points. **(B)** Variations of GSH fluorescence intensity and 4T1 cells survival status after co-culture of CCF and 4T1 cells in different concentrations. Reproduced with permission. Copyright 2021 John Wiley & Sons. **(C)** The schematic illustration of DHA@MIL-101 NRs prompting CDT-ferroptotic cancer therapy through inactivating GPX4 directly or indirectly. **(D)** Comparison of GSH content in Lewis cells after incubation with DHA, MIL-101 NRs, and DHA@MIL-101 NRs. **(E)** The morphology of mitochondria (main characteristic of ferroptosis) when treated with DHA@MIL-101 NRs using TEM. Reproduced with permission. Copyright 2022 BioMed Central. **(F)** Schematic diagram of ferroptosis generated by GSH-deletion mediated GPX4 inactivity. **(G)** Examining GSH depletion capacity of PBS, Mn-TCPP, MOF, and Mn-MOF with different concentrations of Zr. **(H)** Alterations of intracellular GSH content and **(I)** GPX4 activity after treatment of H22 cells with PBS, Mn-TCPP, MOF, and Mn-MOF respectively. Reproduced with permission. Copyright 2021 Ivyspring International Publisher.

In the past few decades, CDT has become an emerging invasive TME-responsive cancer treatment based on Fenton or a Fenton-like reaction, which uses transition metal (including Fe, Mn, and Cu) to convert endogenous abundant H_2_O_2_ into highly toxic •OH, contributing to irreversible cellular damage. As an example, Liu and co-workers designed a Fe^2+^-contained and PEG-modified nanocatalyst reacting with overexpressed H_2_O_2_ to generate sufficient •OH, which is able to kill tumor cells (Huo et al., 2019). Nevertheless, excessive GSH is capable of neutralizing a great deal of ROS, which drastically weakens the metal-mediated Fenton reaction effect (Wang et al., 2022). Considering the characteristics of the tumor environment, the GSH-scavenging strategy is indispensable to relieve the deficiency of •OH, thus prompting the effectiveness of CDT. For instance, Li et al. constructed tumor-specific CaO_2_ nanoparticles encapsulated with Cu-ferrocene for oxidating GSH, named CCF (Kong et al., 2021). Cu^2+^, which released from decomposed CCF in an acidic environment, triggered GSH exhaustion (Figure 5A) and Cu^+^-mediated Fenton-like reaction, avoiding the potential •OH elimination. The intracellular GSH is decreased with the increased CCF concentration, accompanied by increased tumor cell apoptosis (Figure 5B), which demonstrates the antitumor effect of GSH-promoted CDT.

Ferroptosis, a regulatory cell death mode, is characterized by iron-dependent and lipid peroxide (LPO) accumulation, proposed by Dixon et al. (2012). Researchers have revealed that the morphological characteristics of ferroptosis are the diminishment of mitochondrial volume, the augmentation of membrane density, and the disappearance of mitochondria (Chen et al., 2021). Ferroptosis is believed to mainly be the imbalance between oxidative stress and the antioxidant system, thus investigating the mechanism of ferroptosis-promoted tumor cell death triggered by intracellular peroxide accumulation is of great significance. Related studies have proved that the mechanisms of ferroptosis mainly include iron-mediated metabolism disorder and amino acid antioxidant system imbalance (Li et al., 2022). First of all, intracellular iron generally exists in the form of Fe^2+^, which can produce •OH through the Fenton reaction, and then react with polyunsaturated fatty acids in the plasma membrane to produce ROS, resulting in iron-regulated death. Secondly, system X_c_^−^, an antiporter of two catalytic subunits, is always utilized for transporting cystine into cells for GSH synthesis. As a key antioxidant in the body, GSH is also a cofactor of GPX4. Concretely, GPX4 uses GSH as the substrate to reduce phospholipid hydroperoxides (PLOOHs) to hydroxyl derivatives (PLOHs), and oxidizes GSH to GSSH, therefore alleviating the damage of oxidative stress to tumor cells (Stockwell, 2022). Taking account of the mechanisms of ferroptosis, GSH-depleted methods have been extensively exploited for antitumor therapy based on GSH-enhanced GPX4 inactivity and LPO accumulation.

As a case in point, Li and co-workers loaded dihydroartemisinin (DHA) into iron-based MOFs to design a peroxidase-like nanoreactor (termed DHA@MIL-101 NR) for synergistic CDT-ferroptotic therapy (Figure 5C) (Yang et al., 2022a). DHA@MIL-101 NRs self-disassembled in the acid microenvironment to dissociate Fe^3+^, achieving GSH consumption-mediated redox reaction and Fe^2+^-mediated Fenton reaction (Figure 5D). DHA not only reduces GSH to GSSG but also inactivates GPX4 directly, facilitating LPO accumulation and cell ferroptosis collaboratively (Figure 5E). Further to this, Gan et al. introduced Zr into a manganese porphyrin-based MOF (Mn-MOF) to construct a biocompatible nanosensitizer along with the capacity of GSH exhaustion-enhanced ferroptosis (Figure 5F) (Xu et al., 2021). The Zr released from Mn-MOF downregulated GSH efficiently (Figure 5G), thus elevating Mn-MOF-induced ROS concentration, leading to the deactivation of GPX4 and ROS-enhanced ferroptosis. The authors also validated that GSH content and GPX4 activity were significantly decreased when H22 cells were co-incubated with Mn-MOF (Figures 5H, I).

### 4.3 Amplified PDT and SDT

**FIGURE 6:**
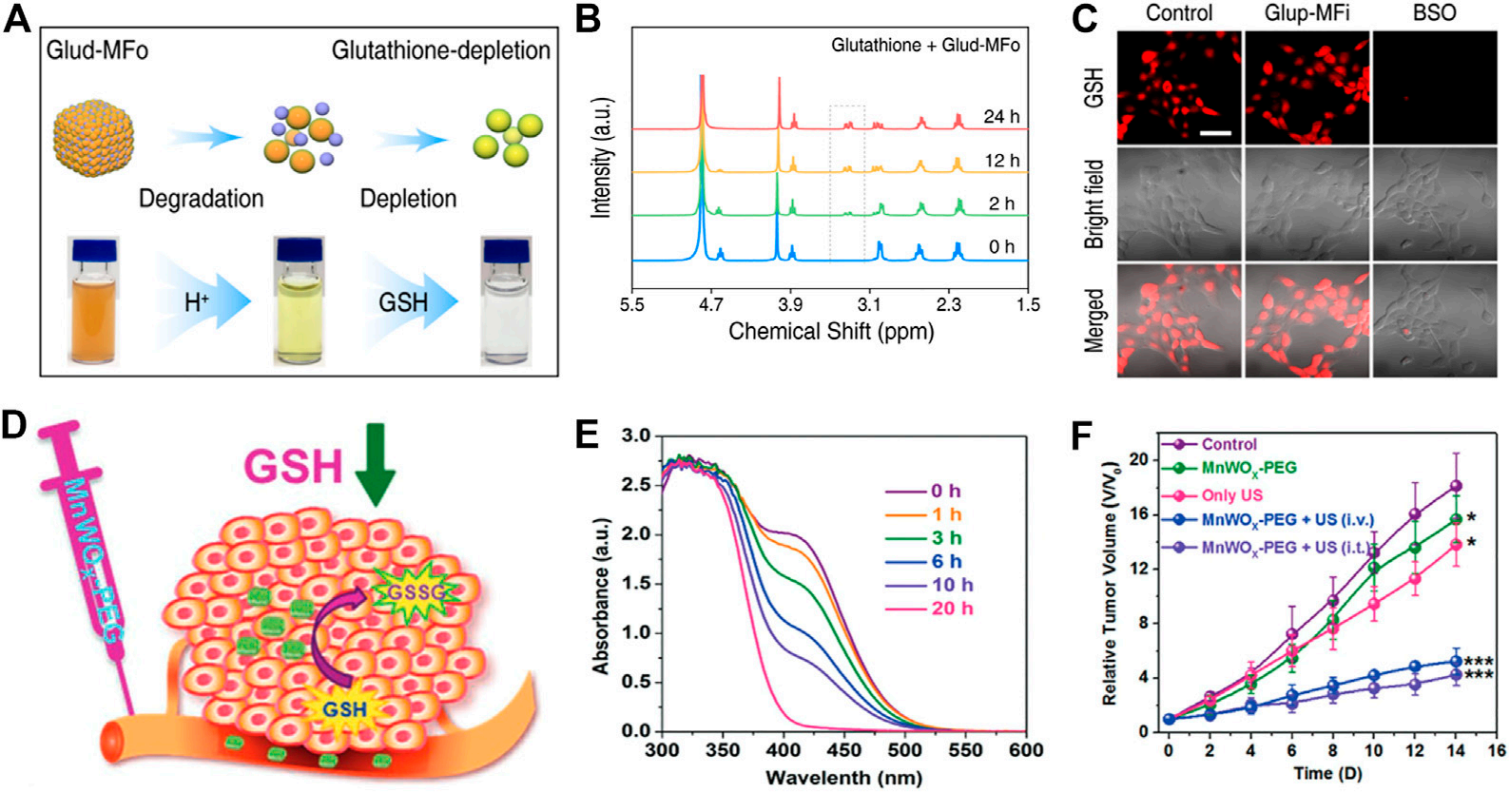
**(A)** Schematic diagram of Glud-MFo-c degradation in an acidic environment. **(B)** Hydrogen-nuclear magnetic resonance spectra (^1^H NMR) of GSH after incubation with Glud-MFo-c at different time points. **(C)** Comparison of GSH content in confocal images after different treatment of 4T1 cells. Reproduced with permission. Copyright 2020 American Chemical Society. **(D)** A diagram of GSH depletion mechanism in tumor tissue. **(E)** The GSH consumption performance of MnWO_X_-PEG at different time points. **(F)** Tumor growth of mice after different treatments. Reproduced with permission. Copyright 2019 John Wiley & Sons.

PDT, a novel cancer treatment method, is appliable to a variety of superficial tumors. Compared with surgery, chemotherapy, and other anticancer means, PDT has multiple advantages such as non-invasiveness, negligible drug resistance, and spatial selectivity. The main therapeutic mechanism of PDT is that photosensitizers were employed to convert oxygen into highly cytotoxic singlet oxygen (^1^O_2_), •OH, and so on under light irradiation, thus causing irreversible damage to tumor tissue. However, hypoxic conditions and high concentrations of reduced GSH in the TME conspicuously compromise the antitumor effect of PDT. In particular, GSH can effectively scavenge ROS and maintain intracellular reductive homeostasis, further promoting the occurrence and development of tumor (Yang et al., 2020; Yang et al., 2022b). Several excellent GSH-responsiveness and -exhaustion nanoagents have been widely introduced to tumor tissue for effective PDT. Recently, a multicomponent self-assembled nanoagent was reported by Zhao and co-workers for GSH consuming-enhanced PDT therapy (Wang et al., 2020b). This nanoagent was self-assembled by photosensitizer Ce6 for ^1^O_2_ production and Fe^3+^ for GSH consumption, encapsulated with polyvinylpyrrolidone (PVP) polymer, named Mn_3_ [Fe(CN)_6_]_2_-Ce_6_ nanoagent (Glud-MFo-c). Interestingly, Glud-MFo-c exhibited a pH-responsive degradation capability in the tumor microenvironment, along with the release of Ce6 and Fe_3_(CN)_6_. With the increased addition of GSH, the color of Glud-MFo-c changed (Figure 6A), suggesting that Fe_3_(CN)_6_ was reduced to Fe_2_(CN)_6_ and GSH was converted to GSSG (Figure 6B). At the same time, the authors also found that GSH content in 4T1 cells was sharply cut down owing to Glud-MFo-c GSH-depletion ability (Figure 6C), which effectively amplified Ce6-produced ^1^O_2_ accumulation, and provided a potential method to combat cancer. Shi et al. proposed a nanocluster, which integrated catalase (CAT) with MnO_2_ to realize CAT-triggered oxygen release and Mn^4+^-assisted GSH exhaustion, accompanied by abundant ^1^O_2_ accumulation, achieving a remarkable anticancer performance characterized by GSH depletion-improved PDT (Zhu et al., 2020).

Instead of applying light and photosensitizers to achieve efficient cancer therapy, SDT employs ultrasound (US) and sonosensitizers to trigger ^1^O_2_ or •OH generation, exerting a crucial role in ablation of tumor tissue that is hard to reach through light irradiation. For instance, Liu et al. designed a US-triggered MnWO_X_ nanosystem modified with PEG (termed MnWO_X_-PEG) to endow the capability of GSH-enhanced ROS (^1^O_2_ and •OH) production for killing malignant tumors (Figure 6D) (Gong et al., 2019). Specifically, MnWO_X_-PEG exhibited significantly time-dependent GSH consumption capacity through reducing W^6+^ to W^5+^ (Figure 6E), cooperating with increased ROS to achieve a powerful inhibition effect on tumors (Figure 6F). Moreover, Lin et al. utilized PEG-modified Bi_2_MoO_6_ to design nanoribbons, named Bi_2_MoO_6_-PEG NRs (BMO NRs), for GSH-enhanced SDT (Dong et al., 2021). BMO NRs not only acted as a piezoelectric nanosensitizer but also as a GSH scavenger. When BMO NRs were incubated with GSH after 12h, Mo^6+^ turned to Mo^5+^ accompanied by the production of GSH-activated BMO NRs (termed GBMO NRs), which displayed a more significantly US-triggered ROS generation effectiveness. In short, the above experiments indicate that the treatment of GSH consumption-enhanced PDT and SDT has a profound effect on retarding tumor progression.

### 4.4 Robust radiotherapy

Radiotherapy (RT) is critical for cancer therapy, and about 50% of patients will receive ionizing radiation during their treatment period (Moding et al., 2013). Radiation-triggered cell damage mainly includes direct and indirect modalities. In general, direct damage refers to the direct injury to DNA induced by ionizing radiation. Indirect damage, accounting for two-thirds of the radiation-induced cell injury, means ionizing radiation is able to react with substances in cells such as water to form toxic ROS (Hall et al., 2016). Nevertheless, as an effective antioxidant, the overexpression of GSH in tumor cells is one of the typical reasons for reducing free radicals, and consequently contributing to radiation resistance (Xie et al., 2019). Therefore, the introduction of nanoparticles with GSH-responsive or -depleted characteristics can effectively sensitize radiotherapy and further significantly improve the anticancer therapeutic effect.

**FIGURE 7:**
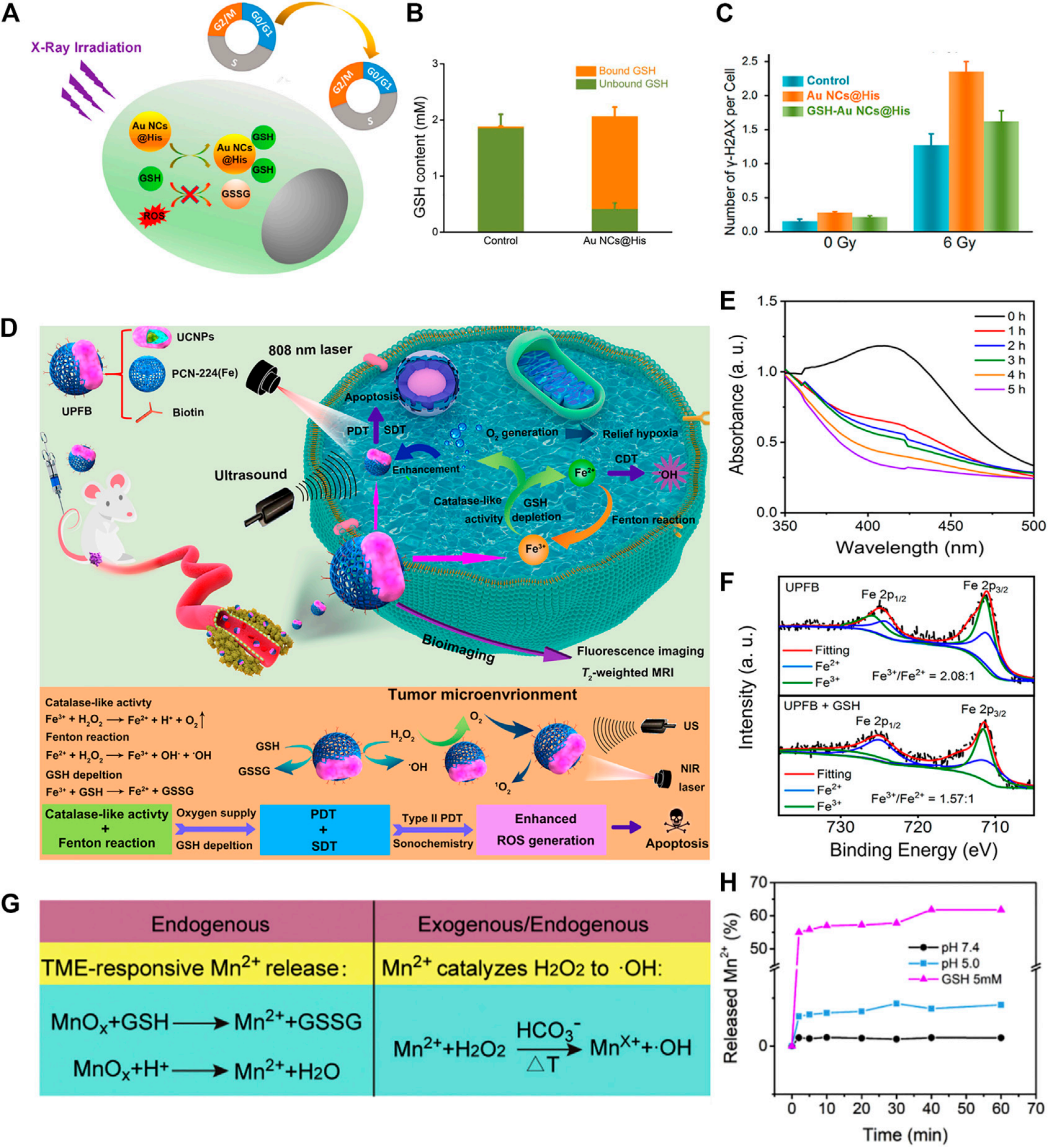
**(A)** Schematic illustration of GSH exhaustion-enhanced radiotherapy with the introduction of Au NCs@His. **(B)** GSH content in the cells without and with Au NCs@His treatment. **(C)** Comparison results of DNA damage molecular (named γ-H2AX) expression degree between Au NCs@His and GSH-Au NCs@His under different doses of radiation. Reproduced with permission. Copyright 2018 American Chemical Society. **(D)** Amplified effect of PDT, CDT, and SDT through GSH depletion and O_2_ supplement for multimodal synergistic therapy. **(E)** GSH depletion ability of UPFB in different incubation time. Reproduced with permission. Copyright 2020 American Chemical Society. **(F)** The proportion of Fe^3+^/Fe^2+^ in the presence or absence of GSH. **(G)** Different antitumor mechanisms of MS-BSA rely on endogenous and exogenous factors. **(H)** Mn^2+^ was released from MS-BSA under reductive and acidic environments. Reproduced with permission. Copyright 2021 Elsevier.

As a paradigm, Wu and co-workers integrated Au nanoclusters (Au NCs) with histidine to accomplish GSH exhaustive-prompted RT, denoted as Au NCs@His (Figure 7A) (Zhang et al., 2018). Besides the intrinsic radiosensitizing property, Au NCs@His can effectively reverse radiation resistance *via* depleting GSH with the ability of forming Au-S bond (Figure 7B). To examine whether GSH consumption is the main reason for radiotherapy improvement, researchers designed another group named GSH-Au NCs@His through mixing GSH with Au NCs@His. Interestingly, the cytotoxicity of 2 NCs assayed in U14 cells showed that Au NCs@His is more toxic than GSH-Au NCs@His. The main reason is that GSH-Au NCs@His does not have the capacity to form an Au-S bond, which greatly reduces the effectiveness of GSH-Au NCs@His to consume GSH, thus protecting tumor cells from ROS oxidative damage (Figure 7C). In addition, radiotherapy sensitizers containing variable valence metals can effectively sensitize radiotherapy *via* oxidation-reduction reactions with GSH. For instance, Gu et al. synthesized nanoclusters possessing multiple high Z elements, such as Fe^3+^ and W^6+^, to give them the ability of remarkable GSH exhaustion and amplified ROS generation upon radiation, both of which have been prevalently exploited as promising anticancer methods (Zhou et al., 2019). As an example, they constructed Sandwich-type polyoxotungstate nanoclusters (termed Fe_4_Se_2_W_18_ NCs) for GSH depletion-sensitized RT. The moderately acidic environment can trigger Fe_4_Se_2_W_18_ NCs to release Fe^3+^, which can be reduced by GSH to produce Fe^2+^ along with the decomposition of H_2_O_2_ for •OH production, rendering cancer cells more susceptible to oxidative stress (Zhou et al., 2021).

### 4.5 Multimodal synergistic therapy

As mentioned above, we clearly recognize that GSH-responsive and -exhausting metal nanomedicines have a prominent effect on cancer therapy. However, the antitumor effect of monotherapy still cannot satisfy the therapeutic needs due to the disadvantages of TME, such as severe hypoxia condition and overexpressed GSH. It is highly desirable to integrate different therapeutic methods together to achieve multimodal synergistic therapy for cancer treatment. Inspired by the diversity of nanoplatforms, synthesis of multifunctional nanoagents with the capability of GSH-responsiveness or -exhaustion have attracted more and more attention in recent years.

For example, Huang et al. fabricated an intelligent nanocatalytic material consisting of copper-doped calcium phosphate (CuCaP) NPs, PEG-modified glucose oxidase (GOx), and the chemotherapeutic drug doxorubicin (DOX) PGC-DOX for efficient cancer therapy (Fu et al., 2021). Once PGC-DOX was swallowed, GOx, Cu^2+^, and DOX will be released in response to acidic environments. GOx, as an enzyme catalyst which catalyzed the conversion of glucose into H_2_O_2_, can produce starvation effect on tumor cells as well as provide raw materials for Fenton-like reactions. Simultaneously, released Cu^2+^ causes redox reaction with the intracellular overexpressed GSH, and subsequently produces Cu^+^ for initiation of Fenton-like reactions, resulting in the production of •OH and robust anticancer therapy. Specifically, the integration of GOx-triggered H_2_O_2_ self-supply, GSH depletion-amplified Fenton-like reactions, and DOX-mediated chemotherapy collaboratively improved the tumor microenvironment, exerting a crucial role in cancer eradication. Furthermore, Quan et al. designed a multi-functional nanoplatform consisting of upconvention nanoparticles (UCNPs) and porphyrin-based MOFs [PCN-224(Fe)], denoted as UPFB (Figure 7D). The nanoparticles can be synchronously driven by near-infrared (NIR) and ultrasound, cooperatively enhancing the anticancer effect of PDT, SDT, and CDT (Wang et al., 2021d). In detail, UPFB can be co-activated *via* laser and US under irradiation to release Fe^3+^. Fe^3+^ can act as a catalase-like nanozyme to transform H_2_O_2_ into O_2_, relieving the hypoxic condition in TME and boosting oxygen-based PDT and SDT. Additionally, Fe^3+^ can be reduced to Fe^2+^ *via* oxidizing GSH, accompanying the production of •OH through a Fenton-like reaction. As expected, when increasing the incubation time with GSH, UPFB displayed a time-dependent GSH consumption capacity (Figure 7E). Simultaneously, the significantly diminished proportion of Fe^3+^/Fe^2+^ ratio further indicated that UPFB was utilized to exhaust GSH through a redox reaction (Figure 7F). Collectively, the combination of treatment based on PDT, SDT, and CDT promoted the accumulation of highly poisonous ^1^O_2_ and •OH, thus producing a drastically powerful anticancer effect. In another case, a two-dimensional (2D) composite nanoagent was prepared by Lin et al. for endogenous and exogenous synergetic cancer therapy (Figure 7G) (Duan et al., 2020). The authors combined KMnO_4_ with bovine serum albumin (BSA)-functionalized silicene to develop a TME- and GSH-responsive nanosheet, denoted as MnOx@silicene-BSA (MS-BSA). This type of nanosheet can be quickly decomposed under overexpressed GSH and an acidic environment (Figure 7H), and can release Mn^2+^ as well as turn GSH into GSSG. Meanwhile, Mn^2+^ catalyzed H_2_O_2_ to produce toxic •OH through Fenton-like reactions under the assistance of HCO_3_^2-^ and high temperatures, which was engineered by an MS-BSA-triggered photothermal effect, potentiating a GSH depletion-prompted robust anticancer effect. Taken together, multimodal synergistic therapy on the basis of nanotechnology does provide revolutionized value and practical application in cancer therapy. However, there are still a variety of challenges to face before entering clinic. Firstly, the synthesis of GSH-depleted multicomponent nanomedicines is so time-consuming that it still faces a huge technological gap from laboratory preparation to industrial production (Klochkov et al., 2021). Secondly, the combination of different functional nanoagents may cause corresponding toxic side effects to the human body, thus the biodegradability, stability, and safety of nanomedicines still need further validation. Moreover, more clinical experimental evidence is required to verify the effectiveness of nanomaterials, so as to develop more GSH-exhaustive and -responsive strategies for targeted cancer therapy.

## 5 Conclusion and outlook

Cancer cells show overexpressed GSH that may maintain redox homeostasis to protect cells from oxidative damage, as well as reduce the chemotherapeutic agent-induced toxification. Thus, depletion of intracellular GSH is considered a potential solution to boost the efficacy of chemotherapy and ROS-based therapy, including PDT, SDT, CDT, ferroptotic therapy, and radiotherapy. Meanwhile, the high levels of GSH in cancer cells can be utilized to design redox-responsive delivery systems for intracellular drug release. Over the past few years, we have seen the vital roles the metal nanomedicines play in the GSH-responsive and -exhausting strategies, and inorganic nanomaterials, MOFs, and platinum-based nanomaterials-mediated GSH depletion is expected to realize more powerful synergistic therapy based on rational design. In this review, we have summarized and outlined the recent advancements, mechanisms, prospects, and challenges of GSH-responsive and -exhausting metal nanomedicines from different strategies. Overall, although rapid progress has been achieved in this field in recent years, some crucial issues and challenges remain to be solved.

(I) The relevant mechanisms involved in each step of the intracellular GSH exhaustion process need to be further explored, and the biological effect of cancer cells after GSH depletion remains to be elucidated. Meanwhile, knowing the mechanism of GSH-responsive and -exhausting cancer treatment strategies based on metal nanomedicines is vital for selecting an optimized approach to GSH consumption and improving the efficiency of cancer therapy.
(II) Despite the intracellular GSH being significantly consumed in the cases stated in this review, it is difficult to completely deplete GSH *via* these strategies. As intracellular GSH is exhausted, the GSH synthesis system will be activated accordingly, which would weaken the efficacy of GSH exhaustion-based cancer therapies in long-term treatment (Niu et al., 2021). Recently, strategies of GSH biosynthesis inhibition connected to various pathways and enzymes have made significant progress for cancer therapy. Upstream cysteine supply block, NADPH control, glutathione reductase activity repression, GSH efflux pumps stimulation, and so on have proven to be feasible approaches to ablate tumors (Xiong et al., 2021). Hence, GSH exhaustion based on multi-strategy therapy would be a desirable solution.
(III) The design of a GSH-responsive and -exhausting drug delivery system can achieve tumor specific ablation to a certain extent. However, off-target effects still cause the risk of normal cell damage and serious side effects. Therefore, more efforts should be taken to develop metal-based nanoplatforms with excellent targeting specificity to cancer cells based on the characteristics of TME and GSH exhaustion mechanisms. On the other hand, it is beneficial to introduce advanced analytical techniques that accurately measure the changes of intracellular GSH/GSSG concentrations.
(IV) The metal nanomedicines show great potential for cancer therapy not only because of the capability of GSH exhaustion, but also for their wide range of other biological effects, such as interference with osmotic pressure, regulation of the immune system, and activation of biocatalysis (Hu et al., 2022). Therefore, a comprehensive exploration and assessment should be performed to elucidate the relation behind each mechanism. Meanwhile, the *in vivo* metabolism and excretion behavior of metal nanomedicines should also be conducted.

Despite the issues and challenges summarized above, we should take full advantage of nanotechnology and overcome these hurdles. The novel and versatile metal nanomedicines with GSH-responsive and -exhausted capability are highly desirable for continuous advance in anticancer research, and this strategy will find more clinical applications in the future.
